# Synchronous metastases from colorectal cancer. Treatment and long-term survival compared to patients with metachronous metastases: a population-based study from Central Norway 2001–2015

**DOI:** 10.2340/1651-226X.2025.42985

**Published:** 2025-06-18

**Authors:** Per Even Storli, Rachel Genne Dille-Amdam, Gaute Havik Skjærseth, Mads Vikhammer Gran, Tor Åge Myklebust, Jon Erik Grønbech, Erling A. Bringeland

**Affiliations:** a Department of Gastrointestinal Surgery, Clinic of Surgery, St. Olavs Hospital, Trondheim University Hospital, Trondheim, Norway; b Department of Clinical and Molecular Medicine, Norwegian University of Science and Technology, Trondheim, Norway; c Department of Registration, Cancer Registry of Norway, Norwegian Institute of Public Health, Oslo, Norway; d Department of Research and Innovation, Møre and Romsdal Hospital Trust, Ålesund, Norway

**Keywords:** Colorectal cancer, synchronous metastases, metachronous metastases, survival, population based

## Abstract

**Background:**

Reliable and modern information on primary evaluation, treatment, and long-term survival rates for patients with colorectal cancer (CRC) metastases are needed. Whether synchronous CRC metastases carry a worse prognosis than metachronous is still debated.

**Methods:**

Population-based study on 7,950 CRC patients from Central Norway, 2001– 2015. Of these, 1,843 (23.2%) had synchronous metastases and of radically operated patients with stage I–III disease 1,117 (20.1%) developed metachronous metastases. The treatment strategies and outcomes for patients with metastases were analyzed, stratified by three consecutive 5-year periods.

**Results:**

Median and 3-year survival for patients with synchronous metastases were 11 months and 16.4%, compared to 17 months and 29.4% with metachronous metastases, *p* < 0.001 and *p* < 0.001, respectively. The subsets receiving supportive care only, had a median survival of 3–4 months. Patients with synchronous metastases and primary palliative chemotherapy had a median survival of 15 months compared to 18 months with metachronous metastases, *p* < 0.001. Neither groups improved survival across the study period. The 5-year survival for the 342/1,843 (18.6%) patients with synchronous metastases and curative intent treatment was 41.8% compared to 43.6% for the corresponding 422/1,117 (37.8%) patients with metachronous metastases, log-rank *p* = 0.281. Survival significantly improved for both these groups across the study period.

**Interpretation:**

A key determinant of better survival for patients with metachronous CRC metastases compared to synchronous was a significantly higher proportion treated with curative intent. Survival for both patients with synchronous and metachronous metastases taken collectively steadily improved during the study period, driven by the increased proportions and improved survival for the subsets with curative intent treatment.

## Introduction

Depending on definition, some 18–25% of colorectal cancer (CRC) patients are diagnosed with synchronous metastases, the proportion seemingly increasing with time [1–3], while 17–23% develop metachronous metastases following radical treatment of CRC stages I–III [3, 4]. Synchronous or early metachronous metastases have been associated with an aggressive biology, thought to confer a poor prognosis [5–7]. Some studies, however, like a spin-off article from a prospective study on colonoscopy [8] and a German trial on sequential chemotherapy in advanced CRC [9] found no survival difference between patients with synchronous and metachronous metastases, possibly due to heavy selection. Population-based studies may grasp a wider picture on incidence, treatment, and prognosis [10–13], but many rely on registries with limited information on, for example, surgery, pathology or chemotherapy [11, 13].

We have recently published a population-based study from Central Norway on some 8,000 CRC patients spanning the 15 years from 2001 to 2015, observing a significant risk reduction for recurrence across the study period for radically resected CRC stages I–III [14]. Moreover, recent studies have shown increased resection rates and improved long-term survival rates for patients with metachronous metastases [5, 15, 16]. The remaining question is whether similar trends apply to synchronous CRC metastases.

By using the aforementioned database of CRCs [14], the aims of this study were to report on the frequency and primary treatment of synchronous metastases and compare this to the CRC patients that developed metachronous metastases. Furthermore, to compare survival rates for patients with synchronous and metachronous CRC metastases. To assess any temporal trends in treatment strategies and outcomes, analyses were stratified by the three 5-year periods spanning the study.

## Material and methods

The study surveyed 680,000 inhabitants in Central Norway (636,000 in 2001–710.000 in 2015), representing 14% of the national population. By using the Cancer Registry of Norway (CRN), 8,683 reports of CRC were identified from January 1, 2001 to December 31, 2015. The Individual Electronic Patient Journals (EPJ) were reviewed for all patients. For the 7,950 patients with a confirmed CRC diagnosis, demographics, tumor variables, treatment, recurrence, and time of death were registered [14]. No systematic information on Kirsten rat sarcoma viral oncogene (KRAS)/B-RAF proto-oncogene (BRAF) mutations, microsatellite stability (MSS) status, or local venous invasion was available. Method of follow-up for radically treated CRC stages I–III was previously detailed [14]. Metastases were considered synchronous if detected within 30 days of the CRC [6], and emergency CRC surgery defined as within 24 h of diagnosis. Metastatic load was quantified as M1a (one organ involved), M1b (more than one organ), or M1c (involving the peritoneum) according to the Union for International Cancer Control (UICC) staging rules for metastatic CRC [17]. Analyses were stratified by the periods 2001–2005, 2006–2010, and 2011–2015. For study purposes, treatment intention was for all patients counted into one of the three categories: best supportive care (BSC), palliative chemotherapy, and curative intent treatment. In real life, assessment of treatment intention was during the later periods centralized to the Multidisciplinary Team (MDT)-meeting at the university hospital of Central Norway [15, 16, 18]. Adjuvant chemotherapy for colon cancer and upfront radiation therapy for rectal cancer adhered to national guidelines [14]. In the first period adjuvant chemotherapy consisted of 5-fluorouacil (5-FU) and folinic acid, with oxaliplatin added from 2006 [19]. In the palliative setting 5-FU monotherapy was since 2002 largely replaced by combination regimens consisting of 5-FU with oxaliplatin- or irinotecan [20, 21]. During the last two periods agents targeting the vascular endothelial growth factor (VEGF; bevacizumab) or epidermal growth factor receptor (EGFR; cetuximab or panitumumab) were increasingly used in conjunction with oxaliplatin- or irinotecan-based combination regimens [22, 23]. In the neoadjuvant setting patients with resectable synchronous or early metachronous metastases increasingly often received combination chemotherapy following early promising results from the European Organisation for Research and Treatment of Cancer (EORTC) trial published in 2008 [24]. Overall, among patients receiving chemotherapy, whether in a primary palliative, adjuvant, or curative setting, the proportion receiving irinotecan or oxaliplatin combination regimens increased from 54% in the initial time period to 80% in the final. Correspondingly, the proportion receiving 5-FU monotherapy decreased from 46% in the initial time period to 20% in the final. Agents targeting VEGF or EGFR were given to 4% in the first time period, 35% in the second and 55% in the final time period. Survival was counted from the date of diagnosing metastatic disease, and administrative censoring date was April 1^st^ 2024. The study was approved by the Regional Ethics Committee (REK 2018/392) and the paper prepared in accordance with the STROBE guidelines [25].

### Statistics

Continuous variables were summarized by median (range) and compared using the Kruskal-Wallis test. Categorical variables were cross-tabulated and compared using the χ^2^-test. Overall survival curves were estimated by the Kaplan-Meier (K-M) method and compared using the log-rank test. In assessing overall survival for the curative intent treatment groups across the three time periods, Cox proportional hazard regression models were used to account for any imbalances in demographic or tumor variables. No test of proportional hazards was done, as modern views advocate hazard ratios (HR) to be interpreted as weighted averages across the follow-up period [26]. Results were reported with 95% confidence intervals (CI). A *p*-value < 0.05 was considered significant. Analyses were done using Stata version 18.5 and SPSS version 29.0.1.

## Results

Of 7,950 CRC patients, 1,843 (23.2%) had synchronous metastases, with no difference across the time periods, *p* = 0.718, Table 1a. Median age was 73 years (21–99). For patients with radically treated CRC stage I–III, 20.1% developed metachronous metastases, reduced from 23.7% in the first time period to 17.1% in the final, *p* < 0.001, Table 1b. Median age was 72 years (27–94) [10]. Several demographic and tumor variables differed significantly between the synchronous and the metachronous groups, Supplementary Table S1.

**Table 1:** Patients with (a) synchronous and (b) metachronous metastases from CRC in Central-Norway 2001–2015, stratified by time period and treatment intention group, n (%).

	Total	2001–2005	2006–2010	2011–2015	p
Patients diagnosed with CRC	7,950	2,424	2,602	2,924	
**(a) Synchronous metastases**	1,843 (23.2)	568 (23.4)	590 (22.7)	685 (23.4)	0.718
Died within 90 days after resection of primary	243 (13.2)	101 (17.8)	66 (11.2)	76 (11.1)	0.001
Best supportive care (with/without res. primary)	532 (28.9)	177 (31.2)	170 (28.8)	185 (27.0)	0.271
Palliative chemotherapy (with/without res. primary)	726 (39.4)	197 (34.7)	257 (43.6)	272 (39.7)	0.087
Curative intent treatment	342 (18.6)	93 (16.4)	97 (16.4)	152 (22.2)	0.009
Curative treatment completed	283 (82.8)	74 (79.6)	75 (77.3)	134 (88.2)	0.184
Curative treatment not completed	59 (17.3)	19 (20.4)	22 (22.7)	18 (11.8)	

Radically treated CRC stage I-III	5,561	1,673	1,834	2,054	
**(b) Metachronous metastases**	1,117 (20.1)	397 (23.7)	369 (20.1)	351 (17.1)	<0.001
Best supportive care	362 (32.4)	147 (37.0)	114 (30.9)	101 (28.8)	0.039
Palliative chemotherapy	333 (29.8)	124 (31.2)	114 (30.9)	95 (27.1)	0.381
Curative intent treatment*	422 (37.8)	126 (31.7)	141 (38.2)	155 (44.2)	0.002
Curative treatment completed	383 (90.8)	116 (92.1)	125 (88.7)	142 (91.6)	0.567
Curative treatment not completed	39 (9.2)	10 (7.9)	16 (11.4)	13 (8.4)	

* Including 22 patients with synchronous and 20 patients with metachronous metastases, initially considered to be in a palliative position, but after chemotherapy considered converted to a curative intent treatment potential.

### Synchronous metastases, primary treatment

The rate of planned CRC surgery (with/without CRC resection), decreased from 63.4% in the first time period to 52.6% in the final. Emergency surgery due to obstruction, perforation or bleeding decreased from 18.8% to 11.1%, *p* < 0.001. Ninety-day mortality was 16.7% following planned surgery and 28.8% following emergency surgery, *p* < 0.001. For the remaining patients, primary treatment is detailed below and in Table 1a.

#### Best supportive care (± resection of the CRC primary)

The proportion was 28.9%, with no significant evolution across the study period, Table 1a. Median age was 83 (46–99) years, significantly higher than the other treatment groups, *p* < 0.001. A total of 39.8% had resection or deviating surgery, significantly reduced across the study period from 55.9% in the first time period to 34.6% in the final, *p* < 0.001. Median survival was 4 months (0–102) and 3-year survival 3.2% (95% CI: 1.9–5.0%), neither improved during the study period, Table 2a.

**Table 2:** Overall survival of patients with (a) synchronous and (b) metachronous metastases from CRC in Central-Norway 2001–2015. Median survival (months), and 3-year survival (%), stratified by time period and treatment group. Operated patients with 90-days mortality excluded.

	Total	2001–2005	2006–2010	2011–2015	p
	Median – 3-year	Median – 3-year	Median – 3-year	Median – 3-year	
**(a) Synchronous metastases**	**11–16.4**	**9**–**13.2**	**11**–**14.7**	**13**–**20.6**	**0.003/ 0.001**
Best supportive care	4–3.2	5–3.4	3–3.5	4–2.7	0.006/ 0.892
Palliative chemotherapy	15–9.2	15–9.1	16–8.9	15–9.6	0.832/ 0.917
Curative intent treatment*					
Curative treatment completed	60–70.7	46–62.2	53–66.7	68–77.6	<0.001/ 0.045
Curative treatment not completed	24–32.2	24–23.8	29–36.4	19–26.1	0.283/ 0.784
(b) Metachronous metastases **	**17**–**29.4**	**14**–**22.4**	**18**–**33.9**	**22**–**32.5**	**0.002/<0.001**
Best supportive care	3–1.9	3–1.4	3–3.5	4–1.0	0.526/ 0.330
Palliative chemotherapy	18–17.7	18–18.5	17–21.1	20–12.8	0.286/ 0.473
Curative intent treatment***					
Curative treatment completed	57–66.1	41–53.4	71–74.4	61–69.0	0.019/ 0.002
Curative treatment not completed	18–20.5	20–20.0	16–25.0	22–15.4	0.851/ 0.815

* Including 22 patients initially considered in a palliative position.

** From diagnosis of metastases to death.

*** Including 20 patients initially considered in a palliative position.

#### Primary palliative chemotherapy (± resection of the CRC primary)

The average proportion was 39.4%, with an increasing trend across the study period, Table 1a. Median age was 68 years (21–92). Demographic and tumor variables are presented in Supplementary Table S2. Patients with liver-alone metastases constituted the largest proportion with 35.4%, followed by liver-and-other at 20.8%. Median survival was 15 months (1–83) and 3-year survival 9.2% (95% CI: 7.0–11.4%), neither improved across the study period, Table 2a. Patients with isolated lung metastases had the best survival outcomes with a median of 30 (18–83) months. A total of 52.2% of the patients had the CRC resected, reduced from 70.6% in the first time period to 37.9% in the final, *p* < 0.001. Median survival following CRC resection was 19 months (3–83) compared to 12 months (1–67) for non-resected, *p* < 0.001. Corresponding 3-year survival rates were 13.5% (95% CI: 10.0–17.0%) and 4.6% (95% CI: 2.4–6.8%), *p* < 0.001.

#### Curative intent treatment

**Figure 1:**
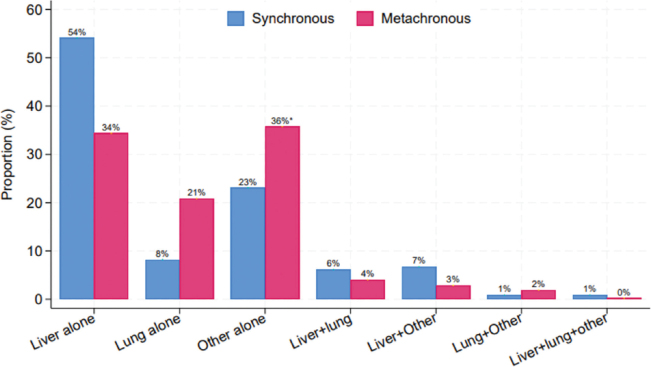
Anatomical distribution of CRC metastases 2001–2015 for patients in the curative intent treatment groups, stratified by synchronous (*n* = 342) and metachronous (*n* = 422) detection. χ^2^ *p* < 0.001. *local recurrences included.

The proportion increased significantly from 16.4% in the first period to 22.2% in the final, including 22 patients converted with chemotherapy, Table 1a. Demographic variables, tumor characteristics, and metastatic load are presented in Table 3 and anatomic distribution of metastases in Figure 1. In particular, 185 patients (54.1%) had isolated liver metastases and 28 patients (8.2%) isolated lung metastases. A total of 73.1% of the patients received neoadjuvant chemotherapy either prior to any surgical intervention or during the interval between CRC surgery and metastatic surgery. The proportion increased from 46.2% in the first time period to 88.8% in the final, *p* < 0.001. A total of 51.5% had liver surgery, 15.5% had lung surgery, and 19.6% had surgery for other metastases, including some with surgery in multiple organs and 16 patients receiving Hyperthermic Intraperitoneal Chemotherapy (HIPEC) treatment. Twenty-two had sustained complete response on chemotherapy and had no resection of metastases.

**Table 3:** Demographic and tumor characteristics compared for the curative intent treatment subgroups of patients with synchronous metastases (*n* = 342) and metachronous metastases (*n* = 422) in Central-Norway, 2001- 2015.

	Synchronous	Metachronous	*p*-value
Median age	65 (29-89)	67 (33-90)	0.040
Sex			0.178
Females	156 (45.6)	172 (40.8)	
Males	186 (54.4)	250 (59.2)	
Primary tumor location			0.135
Right side	89 (26.0)	106 (25.1)	
Left side	128 (37.4)	131 (31.0)	
Rectum	117 (34.2)	168 (39.8)	
Multiple	8 (2.3)	17 (4.0)	
Primary tumor differentiation			0.156
Well (G1)	14 (4.1)	22 (5.2)	
Moderate (G2)	239 (69.9)	318 (75.4)	
Low (G3)	63 (18.4)	61 (14.5)	
Unknown	26 (7.6)	21 (5.0)	
T-stage			<0.001
(y)pT0-1	27 (7.9)	62 (14.7)	
(y)pT2	21 (6.1)	55 (13.0)	
(y)pT3	222 (64.9)	284 (67.3)	
(y)pT4	79 (23.1)	62 (14.7)	
(y)Tx	8 (2.3)	0	
N-stage			<0.001
(y)pN0	107 (31.3)	203 (48.1)	
(y)pN1a-c	114 (33.3)	128 (30.3)	
(y)pN2a-b	105 (30.7)	80 (19.0)	
(y)pNx	16 (4.7)	11 (2.6)	
Metastatic load			<0.001
IVa	261 (76.3)	373 (88.4)	
IVb	42 (12.3)	32 (7.6)	
IVc	39 (11.4)	17 (4.0)	

A total of 59/342 (17.3%) of the patients never completed curative treatment due to, for example, progression during neoadjuvant treatment or during two-stage hepatectomy, Table 1a. Median survival was 24 months (1–74) and 3-year survival 32.2% (95% CI: 21.4–42.9%), with no improvement across the study period, Table 2a. For the *n* = 283/1,843 (15.4%) patients who did complete the curative treatment, recurrence rates were 59/74 (79.7%), 60/75 (80.0%), and 95/134 (70.9%), during the three consecutive time periods. Median and 3-year survival both significantly improved across the study period, Table 2a.

**Figure 2:**
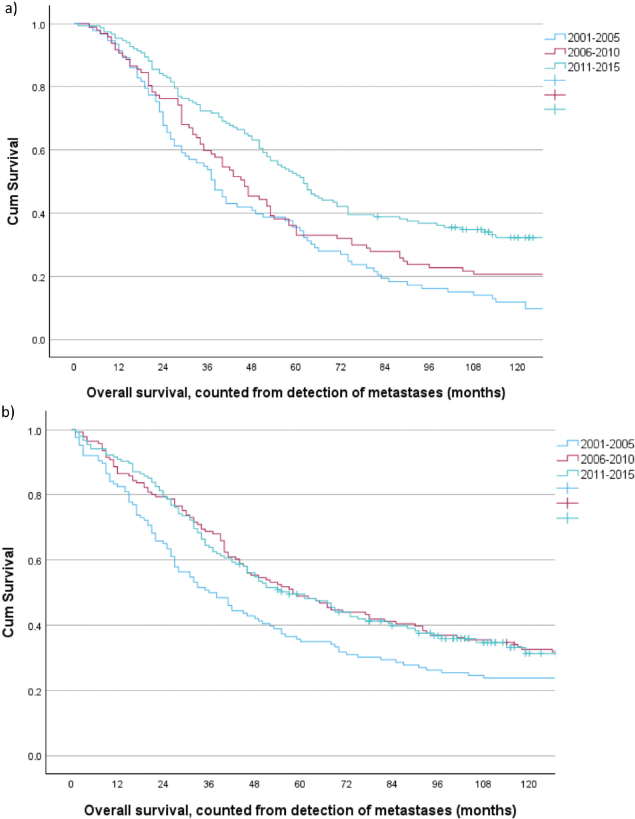
Overall survival for patients with CRC metastases 2001–2015 and curative intent treatment, stratified by time period. (a) synchronous metastases (*n* = 342). Period I versus II, log rank *p* = 0.098, period I versus III, log rank *p* < 0.001, and period II versus III log rank *p* = 0.012. (b) metachronous metastases (*n* = 422), period I versus II, log rank *p* = 0.047, period I versus III, log rank *p* = 0.017, and period II versus III, log rank *p* = 0.997.

For the curative intent group as a whole, 5-year survival improved from 35.5% (95% CI: 25.7–45.3) in the first time period to 52.6% (95% CI: 44.8–60.4) in the last. Survival curves stratified by time period are depicted in Figure 2a, global log-rank *p* < 0.001. Unadjusted HRs for the two last time periods compared to the first were 0.78 (95% CI: 0.57–1.06), *p* = 0.114 and 0.51 (95% CI: 0.38–0.68), *p* < 0.001, respectively. Adjusting for the variables displayed in Table 3, the HRs changed only slightly to 0.79 (95% CI: 0.57–1.09), *p* = 0.158 and 0.47 (95% CI: 0.35–0.64), *p* < 0.001, respectively.

### Metachronous metastases, primary treatment

#### Best supportive care

The average proportion was 32.4% and decreased significantly towards the final period, Table 1b. Median age was 80 (41–94) years, significantly higher compared to the other treatment groups, *p* < 0.001. Median survival was 3 months (0–81) and 3-year survival 1.9% (95% CI: 0.9–3.8%), neither improved during the study period, Table 2b.

#### Primary palliative chemotherapy

The proportion was 29.8% with no significant difference across the study period, Table 1b. Median age was 69 years (27–92). Demographic and tumor variables are presented in Supplementary Table S2. Patients with other-alone metastases formed the largest proportion with 33.0%, followed by liver-alone at 18.6%. Median survival was 18 months (1–101) and 3-year survival 17.7% (95% CI: 14.2–22.5%), neither improved across the study period, Table 2b. Patients with isolated lung metastases had the best survival outcomes, with a median of 28 (1–79) months and 3-year survival at 17.7% (95% CI: 14.2–22.5%).

#### Curative intent treatment

The proportion increased significantly from 31.7% in the first time period to 44.2% in the final, including 20 patients converted with chemotherapy, Table 1b. Demographic variables, tumor characteristics, and metastatic load are shown in Table 3, and anatomic distribution of metastases in Figure 1. In particular, 145 patients (34.4%) had isolated liver metastases and 88 patients (20.9%) had isolated lung metastases. Only 48/422 (11.4%) received neoadjuvant chemotherapy, with no increase across the study period, *p* = 0.991.

A total of 167 (39.6%) patients had liver surgery, 111 (26.3%) had lung surgery and 171 (40.5%) had surgery for ‘other’ metastases, including several with local recurrences and 14 patients that received HIPEC treatment. Eleven patients had sustained complete remission on chemotherapy and was never resected for metastases.

A total of 39/422 (9.2%) did not complete curative treatment due to, for example, progression during neoadjuvant treatment or during two-stage hepatectomy, Table 1b. Median survival was 18 months (3–18) and 3-year survival 20.5% (95% CI: 15.3–25.7%), with no improvement across the study period, Table 2b. For the *n* = 383/1,117 (34.3%) patients that did complete curative treatment, recurrence rates were 77/116 (66.4%), 72/125 (57.6%), and 79/142 (55.6%) in the three respective 5-year periods. Median and 3-year survival, both significantly improved across the study period, Figure 2b.

For the curative intent group as a whole the 5-year survival improved from 35.7% (95% CI: 27.7–44.3) in the first time period to 46.5% (95% CI: 38.7–54.3) in the last. Survival curves stratified by time period are depicted in Figure 2b, global log-rank *p* = 0.049. Unadjusted HRs for the two last time periods compared to the first were 0.71 (95% CI: 0.54–0.94), *p* = 0.017 and 0.57 (95% CI: 0.42–0.77), *p* < 0.001, respectively. Adjusting for the variables displayed in Table 3, the HRs changed only slightly to 0.57 (95% CI: 0.43–0.77), *p* < 0.001 and 0.44 (95% CI: 0.32–0.61), *p* < 0.001, respectively.

### Survival comparing patients with synchronous vs metachronous metastases

**Figure 3:**
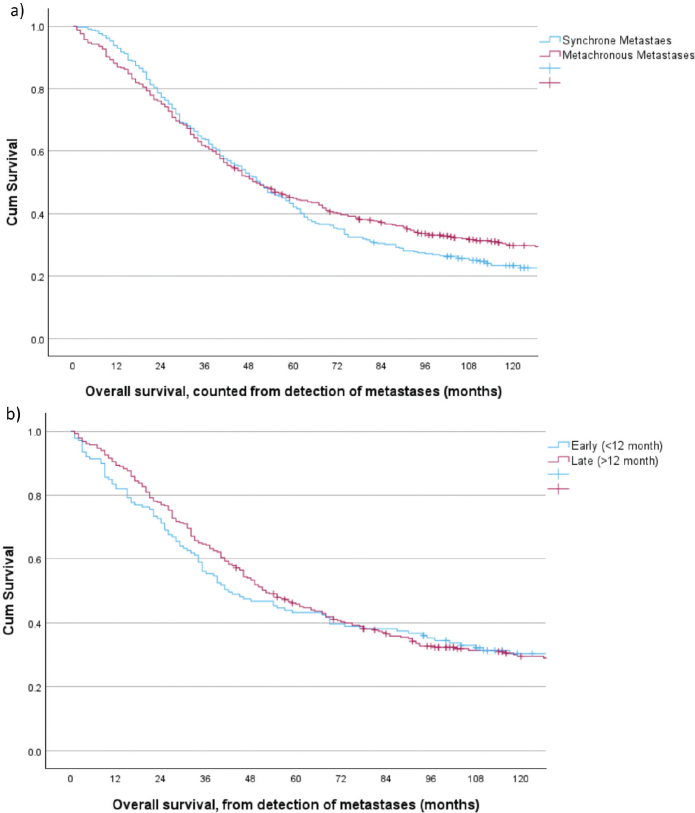
Overall survival for patients with CRC metastases 2001–2015 and curative intent treatment. (a) stratified by synchronous (*n* = 342) versus metachronous (*n* = 422) detection of metastases, log rank *p* = 0.281. (b) stratified by early metachronous (<12 months) and late metachronous (>12 months) detection of metastases, log rank *p* = 0.989.

Taken collectively, median survival for patients with synchronous metastases was 11 months (0–277) compared to 17 (0–269) months for patients with metachronous metastases, *p* < 0.001. The 5-year survival rates were 8.8% (95% CI: 7.6–10.1%) and 17.8% (95% CI: 15.7–20.2%), respectively, log rank *p* < 0.001. However, stratified by the treatment subgroups a more detailed picture emerged. For the BSC-groups no difference in median (*p* = 0.797) or 3-year survival (*p* = 0.401) was found. For the primary palliative chemotherapy groups, median (*p* < 0.001) and 3-year (*p* < 0.001) survival were slightly, but significantly, better for patients with metachronous metastases. Survival curves stratified by time period are depicted in Supplementary Figure S1. For the curative intent groups no difference in survival rates were observed, Figure 3a, nor when dividing the metachronous group into early (≤ 12 months) and late recurrences (> 12 months), Figure 3b.

For the subsets that finally reached a R0/R1 status, median survival was 60 months for patients with synchronous metastases and 57 months for patients with metachronous metastases, *p* = 0.663. Corresponding 5-year survival rates were 50.3% (95% CI: 44.7–56.3) and 48.6% (95% CI: 43.6–53.6), *p* = 0.616.

## Discussion

Taken collectively, survival for patients with synchronous CRC metastases was poor compared to patients with metachronous metastases, replicating findings in a recent Swedish national study [7]. A major determinant for this was the proportion with curative intent treatment that was twice as high in the metachronous group compared to the synchronous. This again reflected that patients with metachronous metastases were a select group proven able to receive and survive radical CRC surgery, whereas patients with synchronous metastases generally suffered from a heavier metastatic load and comprised both old and frail patients, some unable to receive any treatment at all. Hence, comparing survival stratified by the primary treatment intention seems to offer a more informative perspective.

The proportion with curative intent treatment for synchronous as well as metachronous metastases increased across the study period. This followed from expanded resection criteria [15], but also reflects the impact of proper MDT-meetings. A recent Finnish nationwide survey reported resection rates at 36% attributed to centralized and repeated MDT-meetings [16]. This achievement was acclaimed in an editorial as impressive [18]. The percentages of curative intent treatment in this study, excluding the BSC-groups from the denominator as done in the Finnish study, were 32% for synchronous metastases and 56% for metachronous, averaging 42%. This was reached in an environment with centralized MDT-meetings, but with repeated assessments only at the discretion of the treating oncologist.

Assessing time-trends, survival rates have steadily improved for decades both for patients with synchronous and metachronous metastases, whereas conclusions are more cautious for the beginning of the 21st century [7]. In this study survival did improve across the study period for both groups. For patients with synchronous metastases, this was in line with results reported from two European registry studies [11, 13], one of which highlights that the findings were restricted to patients below 60 years [11]. In the present study we found that the improvement was driven by the subset with curative intent treatment, which might serve to explain the reported age restricted survival improvement [11]. For patients receiving primary palliative chemotherapy only, survival did not improve, either for patients with synchronous or metachronous metastases, despite a significant shift towards the use of combination regimen and the addition of targeted agents in the late periods. However, as is evident from Table 1, this finding was confounded. The proportion of patients in the primary palliative chemotherapy groups differed across the time periods since assignments were increasingly shifted from the BSC groups to the palliative chemotherapy groups, concomitant with others shifted from palliative chemotherapy to curative intent treatment following expanded resection criteria. Hence, no lack of improved effect of modern chemotherapy should be inferred.

The reasons for the significantly improved survival rates for the curative intent treatment groups, could be several. As documented, a more restrictive assignment to these groups, artificially inflating survival, was not the case. For the synchronous cohorts the pattern coincided with an increased proportion completing curative treatment in the third period, yet with a substantially reduced recurrence rate. For the metachronous cohort the proportions that completed curative treatment remained stable, while recurrence rates declined significantly from the first to the second period. No differences in demographic variables, tumor characteristics, or metastatic load across the time periods to explain the improved survival was observed (data not shown). An increased use of neoadjuvant combination chemotherapy for patients with synchronous metastases followed in the wake of early promising results reported from the EORTC trial in 2008 [24]. Post or propter, this increase coincided with the improved survival for patients with synchronous metastases and curative intent treatment. However, follow-up data from the EORTC trial tempered the initial optimism when no difference in long-term overall survival rates was found [27]. A recent nationwide Swedish study comparing neoadjuvant chemotherapy to upfront surgery reached the same conclusion [28]. Hence, despite ongoing practice it must be noted, that to date, perioperative chemotherapy has yet through Randomized Controlled Trials (RTCs) to prove its place [27, 29, 30]. Better systemic therapy was considered a pivotal development some decades ago, but has been suggested to play a minor role in further improving survival during the period covered by this study, since no new effective drugs has been introduced since the EGFR-inhibitors in 2007 [7, 22]. Rather, we have previously argued how a parenchymal sparing strategy for liver resections allowed for repeated re-resections in case of recurrent disease [15], and suggest that an important explanatory factor for improved survival may not only reside in the primary treatment delivered, but should also be searched for in the subsequent treatment in case of secondary organ recurrences.

Survival between patients with synchronous and metachronous metastases was compared stratified by treatment intention. For the primary palliative chemotherapy group median survival was 3 months lower for patients with synchronous metastases compared to those with metachronous metastases. Although a small difference, this was statistically significant and at variance with a recent study reporting median survivals of 17.6 and 18.5 months, respectively, *p* = 0.240 [8]. However, the patients had to tolerate at least three courses of chemotherapy and to have their CRC resected, selection criteria that may explain the discrepancy. Survival in this study was also inferior compared to reports from two RCTs [31, 32], however, again, included patients were highly selected and numbers hardly comparable. For the curative intent groups survival rates were similar between patients with synchronous and metachronous metastases. This was consistent with a spinoff article from a German regional screening program [33], where no difference in long-term survival rates were found between patients with synchronous, early metachronous, or late metachronous metastases [9]. Results were, however, at variance with a recent population-based study from Finland, reporting 5-year survival rates at 44% for patients with synchronous metastases, 50% for early metachronous, and 66% for late metachronous [3]. Resection rates were, however, substantially lower and numbers reported were on patients de facto resected and not per treatment intention. In this study, the K-M curves started to separate at 6 years with a continued decline for the synchronous group, possibly due to time-lag bias as patients with metachronous metastases by virtue had their CRC resected at a median 2 years prior to dealing with their metastases. Hence, a follow-up of less than 10 years may not be sufficient to get the full picture. Although the group with synchronous metastases differed from the metachronous group in some variables, we chose to present the unadjusted, monovariable K-M survival estimates when comparing them. This difference represents the everyday clinical situation, which was aimed for to describe. Adjusting for group differences in a Cox regression analysis in the manner done for the time-trend analyses within the cohort of synchronous and metachronous metastases individually, would answer a quite different and more theoretical question.

A final question still lingering for patients with synchronous metastases is any survival benefit of resecting the CRC-primary when in a palliative setting [34, 35]. In this study resection rates were nearly halved from the early period to the last, in line with reported national trends [7, 36]. Although survival was significantly better for those who had their CRC resected, this was likely due to selection bias. The substantially more conservative attitude toward resection of the CRC primary evolving during the study period did not translate into reduced survival rates for the primary palliative chemotherapy group as a whole, in accordance with the only RCT on the subject [37]. A high ninety-day surgical mortality further adds to the sentiment of no benefit of palliative CRC resection unless deemed necessary on clinical grounds.

The clinical implications of a study like this are several. Apart from providing valuable epidemiological information, it supports the contention that most patients with CRC stage IV disease should be considered for evaluation at MDT meetings to identify patients suited for curative intent treatment or with the potential for conversion to curative intent treatment. In addition, the findings suggest that expanded resection criteria and modern oncological treatment keep increasing the number of patients eligible for curative intent surgery, yet maintaining or improving long-term survival rates, indicating that limits may not yet have been reached. Although extending the study inclusion period would have provided more up to date information, detailing the development in the first 15 years of the 21st century is still believed to have its value. The strengths of this study include its large scale, population-based nature, and the documented high compliance from the CRN [38]. Comprehensive data were available from the individual EPJs. Minimum follow-up was 8 years, allowing for few patients to be censored and late recurrences to be registered. The main limitation is its retrospective nature. This could introduce staging bias from improved diagnostic workup over time, although we believe this particular issue to be of subordinate importance as previously detailed [14]. However, retrospectively classifying treatment intentions posed challenges, especially whether to assign patients to the curative intent groups. Formal MDT-meetings spanned the larger part of the study, mitigating this challenge since treatment intentions were now more clearly stated. Furthermore, any initial incongruences between the researcher assessments were resolved by revisiting the EPJs. Finally, the absence of systematic data on KRAS/BRAF mutations, MSS, and local venous invasion is an acknowledged limitation.

## Conclusions

Survival for patients with synchronous CRC metastases was poor compared to those with metachronous, mainly since only 20% received curative intent treatment in the former group compared to 40% in the latter. Stratifying on treatment intention, the observed survival advantage for the metachronous cohort was confined to those receiving primary palliative chemotherapy. For the BSC and the curative intent treatment groups no survival difference between the cohorts with synchronous and metachronous metastases was found. Survival rates for both patients with synchronous and metachronous metastases steadily improved during the first 15 years of the 21st century, driven by the curative intent treatment groups. For the BSC or primary palliative chemotherapy groups, survival did not improve.

## Supplementary Material





## Data Availability

The datasets generated and analyzed during this study are not publicly available due to hospital policy, but are available from the corresponding author on reasonable request.
