# Supplementary material for: Synchronous metastases from colorectal cancer. Treatment and long-term survival compared to patients with metachronous metastases: a population-based study from Central Norway 2001–2015
Source: Acta Oncol. 2025 Jun 18;64:42985. doi: 10.2340/1651-226X.2025.42985 (PMC12186439; doi:10.2340/1651-226X.2025.42985)
Supplement: Supplementary file 1 [file AO-64-42985-s1.pdf]

Supplementary material has been published as submitted. It has not been copyedited, or typeset by Acta Oncologica

Table S1. Demographic and tumour characteristics for all patients with CRC metastases in Central Norway 2001 - 2015, stratified by synchronous (n=1843) and metachronous (n= 1117) detection, n (%).

	Synchronous	Metachronous	p value
**Sex**			0.100
Male	974 (52.8)	625 (56.0)	
Female	869 (47.2)	492 (44.0)	
**Age groups**			0.007
<55 years	165 (9.0)	80 (7.2)	
55- 64 years	339 (18.4)	215 (19.2)	
65- 74 years	470 (25.5)	342 (30.6)	
≥75 years	869 (47.2)	480 (43.0)	
**Primary tumor location**			<0.001
Right colon	756 (41.0)	414 (37.1)	
Left colon	540 (29.3)	306 (27.4)	
Rectum	485 (26.3)	353 (31.6)	
Multiple location	47 (2.6)	44 (3.9)	
Unknown location	15 (0.8)	0	
**Clinical/pathological stage**			*
(y) T0-2 N0	35 (1.9)	106 (9.5)	
(y) T3-T4 N0	206 (11.2)	365 (32.7)	
(y) T0-2 N1-2	19 (1.0)	44 (3.9)	
(y) T3 N1-2	497 (27.0)	419 (37.5)	
(y) T4 N1-2	234 (12.7)	145 (13.0)	
(y) T_{any} Nx	852 (46.2)	39 (3.5)	
**Differentiation**			*
Well (G1)	61 (3.3)	55 (4.9)	
Moderate (G2)	816 (44.3)	767 (68.7)	
Low (G3)	453 (24.6)	233 (20.9)	
Unknown	513 (27.8)	62 (5.6)	
**Metastatic load**			<0.001
Stage IVa	983 (53.3)	799 (71.5)	
Stage IVb	457 (24.8)	196 (17.5)	
Stage IVc	403 (21.9)	122 (10.9)	

* No statistical significance testing due to a high number of entries lacking information

Table S2. Demographic and tumour characteristics for patients with CRC metastases in Central Norway 2001- 2015 receiving primary palliative chemotherapy, stratified by synchronous (n=726) and metachronous (n= 333) detection, n (%).

	Synchronous	Metachronous	p value
**Sex**			0.921
Male	434 (59.7)	198 (59.5)	
Female	292 (40.2)	135 (40.5)	
**Age groups**			0.133
<55 years	94 (12.9)	28 (8.4)	
55- 64 years	178 (24.5)	77 (23.1)	
65- 74 years	244 (33.6)	125 (37.5)	
≥75 years	210 (28.9)	103 (30.9)	
**Primary tumor location**			0.149
Right colon	287 (39.6)	137 (41.1)	
Left colon	200 (27.5)	80 (24.0)	
Rectum	218 (30.0)	102 (30.6)	
Multiple location	16 (2.2)	14 (4.2)	
Unknown location	5 (0.7)	0	
**Clinical/pathological stage**			*
(y) T0-2 N0	4 (0.6)	27 (8.1)	
(y) T3-T4 N0	60 (8.3)	94 (28.2)	
(y) T0-2 N1-2	9 (1.2)	15 (4.5)	
(y) T3 N1-2	190 (26.2)	134 (40.2)	
(y) T4 N1-2	106 (14.6)	55 (16.5)	
(y) T_{any} Nx	357 (49.2)	8 (2.4)	
**Differentiation**			*
Well (G1)	26 (3.6)	12 (3.6)	
Moderate (G2)	313 (43.1)	224 (67.3)	
Low (G3)	188 (25.9)	80 (24.0)	
Unknown	199 (27.4)	17 (5.1)	
**Metastatic load**			<0.001
Stage IVa	316 (43.5)	193 (58.0)	
Stage IVb	236 (32.5)	90 (27.0)	
Stage IVc	174 (24.0)	50 (15.0)	

* No statistical significance testing due to a high number of entries lacking
