# Supplementary material for: Synchronous metastases from colorectal cancer. Treatment and long-term survival compared to patients with metachronous metastases: a population-based study from Central Norway 2001–2015
Source: Acta Oncol. 2025 Jun 18;64:42985. doi: 10.2340/1651-226X.2025.42985 (PMC12186439; doi:10.2340/1651-226X.2025.42985)
Supplement: Supplementary file 2 [file AO-64-42985-s2.pdf]

Supplementary material has been published as submitted. It has not been copyedited, or typeset by Acta Oncologica

Figure S1. Overall survival for patients with synchronous or metachronous CRC metastases 2001 – 2015, receiving primary palliative chemotherapy only, stratified by 5-year periods. a) 2001-2005, log-rank p= 0.051, b) 2006-2010, log-rank p= 0.003, and c) 2011-2015, log-rank, p= 0.094. Global log-rank, p<0.001

a)

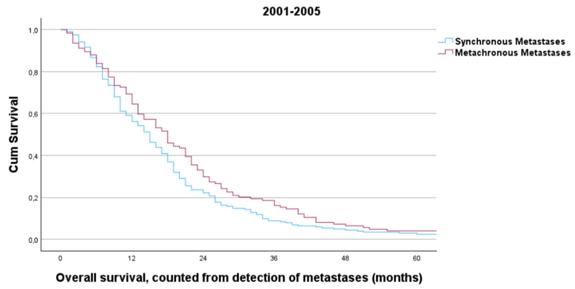

Number at risk

Synchronous	197	113	44	17	9	5
Metachronous	124	79	36	19	7	4

b)

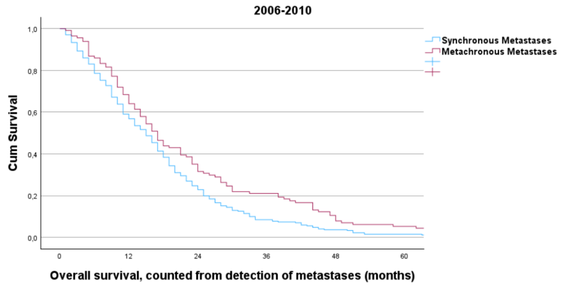

Number at risk

Synchronous	257	150	61	22	9	3
Metachronous	114	72	35	23	8	5

c)

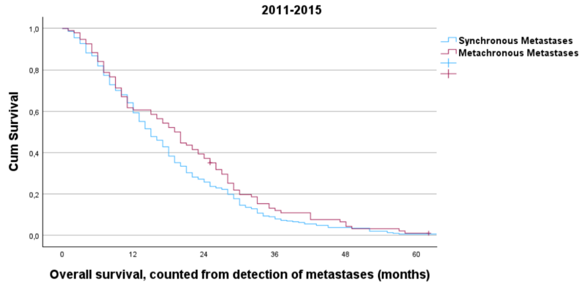

Number at risk

Synchronous	272	166	73	22	10	1
Metachronous	95	56	34	10	3	0
